# Neutrophil Extracellular Traps (NETs) Promote Non-Small Cell Lung Cancer Metastasis by Suppressing lncRNA MIR503HG to Activate the NF-κB/NLRP3 Inflammasome Pathway

**DOI:** 10.3389/fimmu.2022.867516

**Published:** 2022-05-30

**Authors:** Yong Wang, Fen Liu, Lin Chen, Chen Fang, Shuangyan Li, Shangkun Yuan, Xiaoying Qian, Yan Yin, Biao Yu, Biqi Fu, Xinwei Zhang, Yong Li

**Affiliations:** ^1^Department of Medical Oncology, The First Affiliated Hospital of Nanchang University, Nanchang, China; ^2^Medical Innovation Center, The First Affiliated Hospital of Nanchang University, Nanchang, China; ^3^Critical Care Medicine, The First Affiliated Hospital of Nanchang University, Nanchang, China; ^4^Department of Internal Neurology, The Second Affiliated Hospital of Nanchang University, Nanchang, China; ^5^Department of Pathology, The First Affiliated Hospital of Nanchang University, Nanchang, China; ^6^Department of Rheumatology, The First Affiliated Hospital of Nanchang University, Nanchang, China

**Keywords:** neutrophil extracellular traps (NETs), NOD-like receptor protein 3 (NLRP3), MIR503HG, epithelial to mesenchymal transition (EMT), non-small cell lung cancer (NSCLC)

## Abstract

Neutrophil extracellular traps (NETs) that are produced in the tumour microenvironment (TME) have been suggested to play an essential role in the dissemination of metastatic cancer under multiple infectious and inflammatory conditions. However, the functions of NETs in promoting non-small cell lung cancer (NSCLC) metastasis and the underlying mechanisms remain incompletely understood. Here, we found that NETs promoted NSCLC cell invasion and migration by inducing epithelial to mesenchymal transition (EMT). To explore how NETs contribute to NSCLC metastasis, microarrays were performed to identify substantial numbers of long noncoding RNAs (lncRNAs) and mRNAs that were differentially expressed in NSCLC cells after stimulation with NETs. Interestingly, we observed that the expression of lncRNA MIR503HG was downregulated after NETs stimulation, and ectopic MIR503HG expression reversed the metastasis-promoting effect of NETs *in vitro* and *in vivo*. Notably, bioinformatics analysis revealed that differentially expressed genes were involved in the NOD-like receptor and NF-κB signalling pathways that are associated with inflammation. NETs facilitated EMT and thereby contributed to NSCLC metastasis by activating the NF-κB/NOD-like receptor protein 3 (NLRP3) signalling pathway. Further studies revealed that MIR503HG inhibited NETs-triggered NSCLC cell metastasis in an NF-κB/NLRP3-dependent manner, as overexpression of NF-κB or NLRP3 impaired the suppressive effect of MIR503HG on NETs-induced cancer cell metastasis. Together, these results show that NETs activate the NF-κB/NLRP3 pathway by downregulating MIR503HG expression to promote EMT and NSCLC metastasis. Targeting the formation of NETs may be a novel therapeutic strategy for treating NSCLC metastasis.

## Introduction

Non-small cell lung cancer (NSCLC), a common malignant tumour, is the leading cause of cancer-related death worldwide (1). Despite the application of various innovative therapeutic strategies, such as targeted therapy and immunotherapy, to treat NSCLC, the 5-year survival rate of NSCLC patients remains unsatisfactory (2). Among the main reasons for this are high rates of recurrence and metastasis after comprehensive treatment, in particular, after surgical resection (3). Therefore, understanding the detailed molecular mechanism underlying NSCLC metastasis is imperative for improving the quality of treatment and prolonging survival time.

The tumour microenvironment (TME) is composed of various cellular components, such as inflammatory and immune cells, and noncellular components, including the extracellular matrix (ECM), and the TME plays a crucial role in the spread of metastatic cancer (4, 5). Neutrophils, which are the most abundant immune cells, play an essential role in inflammatory responses but are reported to function as tumour accomplices that contribute to the progression and metastasis of cancer (6–8). Neutrophil extracellular traps (NETs), extensive extracellular web-like structures produced and released by activated neutrophils, are composed of modified decondensed chromatin and granule proteins and play an especially crucial role in recognizing and removing pathogens during host defence (9–12). Over the years, NETs induced by inflammation, surgical stress, or cancer cells have been found to accelerate tumour progression by promoting metastasis or forming cancer-associated thrombosis, and these findings have revealed the cancer-promoting function of NETs (13–17). Studies have suggested that NETs facilitate the spread of metastatic tumour cells and their colonization of host tissues by catching circulating tumour cells (CTCs) and accelerating angiogenesis (18, 19). Neutrophil infiltration and NETs formation in lung cancer patient tissues have been described, suggesting that NETs may play an important role in tumour progression (20, 21). NETs have been revealed to play pathophysiological roles in NSCLC progression and metastasis in several studies (18, 22, 23). Despite these findings, little is known about the molecular mechanisms underlying the promotion of NSCLC metastasis by NETs.

Long noncoding RNAs (lncRNAs) are defined as a novel class of transcripts that are over 200 nucleotides in length and have limited or no protein-coding potential (24). In recent studies, multiple pathophysiological diseases processes have been closely related to the dysfunction or abnormal expression of lncRNAs (25, 26). LncRNAs are involved in various cellular processes and regulate multiple cancer-related factors, such as genome stability, cell cycle, growth and immortality, apoptosis, progression, metastasis, and angiogenesis (27). At present, it is not clear whether NETs affect the expression of lncRNAs in tumour cells and promote the metastasis of tumour cells by regulating lncRNA expression. In our previous study, microarrays were used to explore the differential expression profiles of lncRNAs and mRNAs in NSCLC cells after stimulation with NETs (28, 29). Substantial numbers of differentially expressed lncRNAs and mRNAs were identified in NSCLC cells with and without NETs treatment. However, little is known about the role of NETs in NSCLC metastasis and the detailed mechanism by which NETs regulate the expression of specific lncRNAs.

Inflammation is closely related to cancer, and metastasis is complicated by inflammation (29). NOD-like receptor pyrin domain-containing 3 (NLRP3), the most well-characterized and well-studied inflammasome complex, is usually activated by a diverse range of ‘danger signals’, and substantial evidence suggests that the NLRP3 inflammasome exerts a significant effect on the pathogenesis, development, and progression of a variety of tumours, including lung cancer, breast cancer, colon cancer and hepatocellular carcinoma (30, 31). To date, numerous studies have shown that lncRNAs regulate various physiological and pathological processes by targeting the NLRP3 inflammasome (32). Recent studies have also suggested that NETs can promote the pathological process of multiple diseases, including diabetes, autoimmune disease, and inflammation-related disease, by activating the NLRP3 inflammasome (33–35). However, the role of the NLRP3 inflammasome in the effect of NETs on promoting NSCLC metastasis is largely unclear.

Using bioinformatics analysis, we found that the expression of lncRNA MIR503HG was significantly downregulated, whereas overexpression of MIR503HG reversed the metastasis-promoting effect of NETs. NETs activated the nuclear factor-κB (NF-κB) and NOD-like receptor signalling pathways and facilitated epithelial to mesenchymal transition (EMT), thereby contributing to NSCLC metastasis. Further study revealed that NETs promoted NSCLC metastasis by regulating lncRNA MIR503HG expression to facilitate NF‐κB/NLRP3 signalling pathway activation, and lncRNA MIR503HG and NLRP3 may be new targets for the treatment of NSCLC.

## Materials and Methods

### Population and Specimens

The cancer tissues and adjacent noncancerous lung tissues of 50 NSCLC patients were acquired from the pathological specimen repository of The First Affiliated Hospital of Nanchang University, China. Time to metastasis was calculated based on the date of initial treatment to the date of investigator-assessed radiographic organ or node metastasis. The data were censored at the last follow-up or when patients died without metastasis.

### Cell Culture and Animal Study

The human bronchial epithelial cell lines (BEAS-2B) and human NSCLC cell lines A549 and SK-MES-1 were obtained from the Type Culture Collection of the Chinese Academy of Sciences (Shanghai, China). Both cell lines were grown in high-glucose Dulbecco’s modified Eagle’s medium (DMEM, BI, Israel) supplemented with 10% foetal bovine serum (FBS, Gibco, Grand Island, USA) and 1% penicillin and streptomycin solution (Solarbio, China) at 37°C in a 5% CO_2_ humidified atmosphere.

The Ethics Committee of the Medical Innovation Center of the First Affiliated Hospital of Nanchang University approved the animal experimental protocol. Eight-week-old female SD rats, and four-week-old female BALB/c nude mice were purchased from Hunan SJA Laboratory Animal Co., Ltd. The mice were fed under specific pathogen-free (SPF) conditions in accordance with the regulations of the institution.

### Neutrophil Isolation

Neutrophils were isolated from the peripheral blood of healthy donors and SD rats with the peripheral blood neutrophil extraction kit (Solarbio, China). Isolated primary neutrophils were maintained in RPMI 1640 medium (BI, Israel) supplemented with 10% FBS. Giemsa staining and trypan blue viability assays were utilized to determine neutrophil purity (> 98%) and vitality (> 95%).

### Formation and Visualization of NETs

Neutrophils were plated and allowed to adhere in 6-well plates for 1 h before treatment with 100 nM phorbol-12-myristate-13-acetate (PMA, Sigma, USA) for 4 h at 37°C in 5% CO_2_; this treatment allowed NETsosis to occur. Then, based on the protocol recommended by previous studies (36), NETs were harvested following a multistep centrifugation protocol. For quantification, equivalent numbers of neutrophils (1×10^7^/well) grown in 6-well plates were stimulated with PMA (100 nM) for 4 h to generate NETs. Then, the supernatants were slowly and gently extracted and washed twice to remove impurities without disrupting the NETs. The supernatants containing NETs were collected and centrifuged to purify the NETs, which were finally stored at −80°C for subsequent experiments.

Paraffin-embedded lung tissues from NSCLC patients were cut into 5-μm-thick sections for Immunofluorescence assays. The paraffin sections were deparaffinized and rehydrated. The sections were heated in Tris/EDTA buffer (pH 9.0) for antigen retrieval. Isolated neutrophils were seeded on coverslips in 24-well plates. Then, both cells and thin sections of lung tissues were fixed, permeabilized, blocked and stained with primary antibodies against citrullinated histone H3 (cit-H3) (1:250, Abcam, ab5103, UK), myeloperoxidase (MPO) (1:50, Abcam, ab90810, UK) or Ly6g (1:100, Abcam, ab25377, UK) overnight at 4 °C with shaking, followed by incubation with secondary antibodies conjugated to Alexa Fluor 488 (1:200, Elabscience, China) and Alexa Fluor 594 (1:200, Elabscience, China) for 1 h at 37°C. The nuclei were stained with 4′,6-diamidino-2-phenylindole (DAPI, Boster Biological Technology, China) for 5 min. Neutrophil-produced NETs were imaged by fluorescence microscopy (Zeiss) or confocal laser scanning microscopy (Leica) to assess their components. The colocalization of NETs with cit-H3, MPO, Ly6g or DNA was observed. The quantification of NETs was analyzed using Image J software.

### Microarray Analysis

Twelve hours after NETs stimulation, cells were treated with TRIzol (Invitrogen, Carlsbad, USA) and then sent to OE Biotech Co., Ltd. (Shanghai, China) for transcriptome RNA microarray analysis. A |log2 (fold change)| ≥ 2 and *P* < 0.01 were used as the thresholds to determine whether lncRNA expression was upregulated or downregulated. Volcano plots and heatmaps showing differential lncRNAs were generated with R soft and related Bioconductor packages. The top 20 upregulated signalling pathways were identified by Kyoto Encyclopedia of Genes and Genomes (KEGG) analyses.

### *In Vivo* Tumour Metastasis Assay

MIR503HG-overexpressing and empty vector A549 cells were stimulated with NETs the day before the mouse metastasis model was established. These two kinds of A549 cells (2 × 10^6^/mouse) were injected into BALB/c nude mice through the tail vein. After 8 weeks, the mice were euthanized, and the lungs were collected and subjected to H&E and histological staining. Metastasis burden, number of metastasized tumours, volume, and maximum size were evaluated.

### Immunohistochemistry

Paraffin-embedded lung tissues from the above mouse model were cut into 5-μm-thick sections for immunohistochemical (IHC) analysis. Briefly, the paraffin sections were deparaffinized and rehydrated. The sections were heated in Tris/EDTA buffer (pH 9.0) for antigen retrieval and incubated in 3% hydrogen peroxide for 10 min at room temperature to block endogenous peroxidase activity. For IHC staining, the primary antibodies anti-N-cadherin (1:50, Abcam, ab76011, UK), anti-E-cadherin (1:200, Proteintech, 20874-1-AP, China), and anti-vimentin (1:200, Abcam, Ab92547, UK) were incubated with the tissue samples overnight at 4°C. Images of the IHC-stained slides were visualized and analysed at 100× and 400× magnification utilizing an ordinary optics microscope (Zeiss).

### Immunofluorescence Assays

A549 and SK-MES-1 cells were seeded on coverslips in 24-well plates overnight. After preprocessing, the cells were fixed with 4% paraformaldehyde (Solarbio, Beijing, China) for 20 min and permeabilized with 0.5% Triton X-100 (Solarbio, China) for 15 min at room temperature. After blocking with 5% goat serum for 1 h at room temperature, the cells were incubated with primary antibodies against NLRP3 (1:50, Proteintech, 19771-1-AP, China), Caspase1 (1:50, Proteintech, 22915-1-AP, China), and p50 (1:200, Cell Signaling Technologies, 13586, USA) overnight at 4°C with shaking. The cells were incubated with Alexa Fluor 488-conjugated secondary antibodies (1:200, Elabescience, China) for 1 h at 37 °C. The nuclei were stained with DAPI (Boster Biological Technology, China) for 5 min. The samples were visualized by fluorescence microscopy (Zeiss) or confocal laser scanning microscopy (Olympus).

### Reactive Oxygen Species Assay

ROS in A549 and SK-MES-1 cells were evaluated by using the ROS assay kit (Elabscience, China). Briefly, NSCLC cells after preprocessing were incubated with 10 µM 2,7-dichlorodihydrofluorescein diacetate (DCFH-DA) protected from light at 37°C for 30 min, and then followed by washing with PBS to remove excess fluorescence probe. The active cell nucleus was stained using Hoechst 33342. Fluorescence microscopy (Zeiss) was applied to test the fluorescence intensities in NSCLC cells.

### Enzyme-Linked Immunosorbent Assay

The human IL-1β and IL-18 ELISA kit (Boster Biological Technology, China) was performed to assess the level of IL-1β and IL-18 in NSCLC cells supernatant according to the manufacturer’s instructions. Brief, the culture medium was collected after the removal of cell debris by centrifugation at a speed of 1000×g for 10 min. The level of IL-1β and IL-18 in supernatants were analyzed by ELISA kit. Absorbance at 450 nm was detected with a microplate reader (ThermoScientific).

### Subcellular Fractionation

NE-PER Nuclear and Cytoplasmic Extraction Reagents (Thermo Fisher Scientific, 78833, USA) were used to determine the MIR503HG location in A549 and SK-MES-1 cells. According to the instructions of the kit, briefly, dry cells were collected in 1.5-mL microcentrifuge tubes (1-10×10^6^ cells/tube) and washed with PBS, and ice-cold mixture that contained Cytoplasmic Extraction Reagent (CER I), CER II and Nuclear Extraction Reagent (NER) (200:11:100 µL) was added. The tubes were vigorously vortexed at the highest setting and centrifuged at 16,000 ×g for 5 min; then, the supernatant was transferred to a new tube. The above steps were repeated once more, and the extracts were obtained. All the steps were performed on ice, and then, the samples were purified using the FastPure^Ⓡ^ Cell/Tissue RNA Isolation Kit V2 (Vazyme, Nanjing, China) following the manufacturer’s protocol to isolate the nuclear and cytoplasmic RNA. GAPDH (predominantly in the cytoplasm) and U6 (enriched in the nucleus) were used as controls.

### Transient Transfection

A549 and SK-MES-1 cells were seeded in dishes at an appropriate density and cultured in a 37°C incubator overnight. The cells were transfected with plasmids and siRNAs with Hieff Trans liposomal transfection reagent (Yeasen, Shanghai, China) according to the manufacturer’s instructions. The cells were collected 24-48 h after transfection for Western blotting or qRT-PCR analysis.

The NLRP3 expression plasmid was purchased from Vigene Biosciences (Shandong, China). The p50-pcDNA3.1 plasmids were generated by inserting the full-length sequence of p50 cDNA, which was amplified by PCR, into the pcDNA3.1 vector, and then, the constructed p50-pcDNA3.1 plasmid was sent to Genewiz Biotechnology Co. Ltd. (Suzhou, China) for DNA sequencing. siRNAs targeting p50, MIR503HG and the negative controls were designed and synthesized by Gemar Pharmaceutical Technology Co. Ltd. (Shanghai, China). The sequences are as follows:

p50-siRNA-1: 5′-GCUAUAAUCCUGGACUCUUTT-3′;

p50-siRNA-2: 5′-GCAAUCAUCCACCUUCAUUTT-3′.

MIR503HG-siRNA-1: 5′-CCUCUCCCACCAUUUCUUUTT-3′;

MIR503HG-siRNA-2: 5′-GACAAGAACUAAAGUGGAATT-3′;

### Quantitative Real-Time Polymerase Chain Reaction

Total RNA was harvested using the FastPure^Ⓡ^ Cell/Tissue Total RNA Isolation Kit V2 (Vazyme, Nanjing, China) following the manufacturer’s protocol. Real-time quantitative polymerase chain reaction (RT-qPCR) analyses were conducted utilizing a SYBR Green Kit (TransGen Biotech, Beijing, China). The 20-μL qRT-PCR mixture included 10 μL of 2× PCR master mix, 1 μL of 10 μM primers, 7 μL of RNase-free water, and 2 μL of the reverse-transcription template. The qRT-PCR amplification conditions were as follows: 95°C for 10 min, followed by 40 cycles at 95°C for 5 s and 60°C for 30 s. Human GAPDH was utilized as an internal control. The sequences of the PCR primers are provided in **Supplementary Table 1**.

### Western Blotting Analysis

Equal amounts of proteins were used for sodium dodecyl sulfate-polyacrylamide gel electrophoresis (SDS-PAGE). The proteins were transferred to a polyvinylidene fluoride membrane (PVDF, Merck Millipore, Darmstadt, Germany). The membranes were incubated with 5% buttermilk for 1 h at 25°C and then incubated with primary antibodies at 4°C overnight with gentle shaking. The primary antibodies included anti-N-cadherin (ab76011), anti-Vimentin (ab92547), anti-cit-H3 (ab5103), (1:1000, Abcam, UK), anti-NLRP3 (19771-1-AP), anti-E-cadherin (20874-1-AP), anti-Caspase1 (22915-1-AP), anti-GAPDH (60004-1-Ig) (1:1000, 1:5000, 1:1000, and 1:20000 Proteintech, China), anti-p50 (1:1000, Cell Signaling Technologies, 13586, USA), anti-p-p50 (sc-271908), anti-p65 (sc-8008), anti-p-p65 (sc-136548) (all at 1:500, Santa Cruz Biotechnology, USA), and anti-Histone H3 (1:1000, ImmunoWay Biotechnology, YT2163, USA). The next day, the membranes were incubated with horseradish peroxidase-conjugated secondary antibodies specific for rabbit or mouse primary antibodies (Proteintech, China) at a dilution of 1:2000 at 25°C for 1 h. The protein bands were detected utilizing ECL Detection Reagents (Proteintech, China).

### Transwell Invasion and Wound Healing Assay

For the cell invasion assay, the upper chamber of the Transwell 24-well plates (8 μm pores, Corning, NY, USA) was coated with 5% Matrigel (Corning, NY, USA). A549 and SK-MES-1 cells (6×10^4^) suspended in 200 µl of serum-free medium were plated in the upper chamber after treatment. The lower chambers were filled with 600 μL of culture medium supplemented with 10% FBS. The NSCLC cells were permitted to invade through the Matrigel for 48 h, and the cells on the bottom side of the membrane were fixed using 4% paraformaldehyde, the cells on the upper surface were removed with cotton swabs, and then, the invaded cells were with crystal violet. The numbers of invaded cells were counted in five random fields under an optical microscope with ImageJ software.

For wound healing assays, treated cells were incubated in 6-well culture plates and grown to approximately 90% confluence. Scratches were manually established in the cell monolayers with a P200 pipette tip, which guaranteed that all wound widths were consistent. PBS was used to remove cell debris, and then, the scratched cells were cultured in medium supplemented with 1% serum that eliminated the effects of cell proliferation on migration. The wounds in three random fields were photographed at 0 h and 36 h, and the wound widths were measured with ImageJ software.

### Statistical Analysis

The data are shown as the mean ± standard deviation (SD). Statistical analysis was performed using Student’s t test or one-way analysis of variance (ANOVA) to compare the means between two groups or multiple groups, respectively, followed by the nonparametric Wilcoxon rank sum test. The risk of NSCLC patient metastasis was determined with the Kaplan-Meier method. All the analyses were performed using GraphPad Prism 8.0.2 software (San Diego, CA, USA). *P* < 0.05 was considered to be statistically significant.

## Results

### The Release of NETs by Neutrophils Promotes the Metastasis of NSCLC *In Vitro*

**Figure 1:**
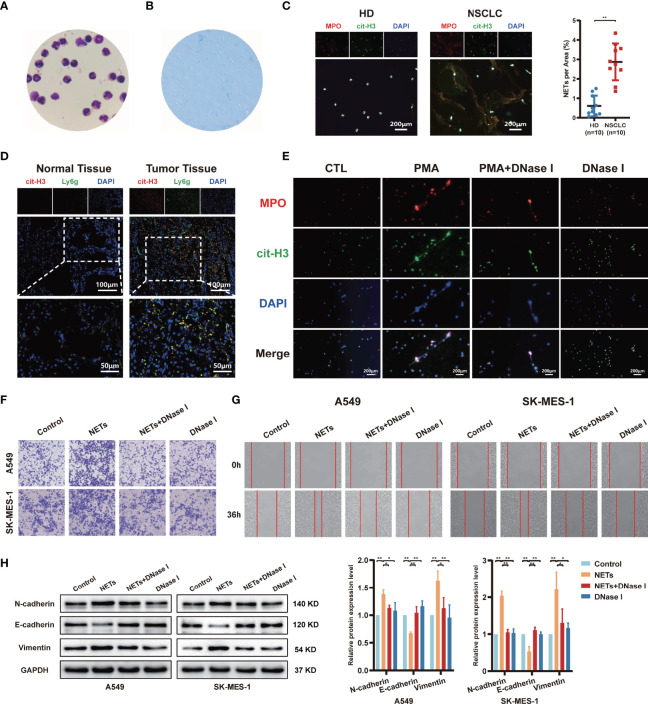
The release of neutrophil extracellular traps (NETs) by neutrophils promotes migration and invasion of NSCLC. **(A)** The morphology of neutrophils isolated from healthy donors’ blood was observed by Giemsa staining (magnification, 1000×). **(B)** The neutrophil viability was assessed by trypan blue dye exclusion assays (magnification, 100×). **(C)** Representative images and quantification of NETs formation of neutrophils from healthy donors (HD) and NSCLC patients. MPO (red), cit-H3 (green), and DAPI (blue), respectively (magnification, 50×; scale bar, 200μm). **(D)** Representative images of NETs formation in NSCLC patients’ normal lung tissues and tumor tissues that were detected by con-focal microscopy. cit-H3 (red), Ly6g (green), and DAPI (blue), respectively (magnification, 200×; scale bar, 100µm and magnification, 400×; scale bar, 50µm). **(E)** Representative images of PMA-induced NETs formation of neutrophils from HD stained with MPO and cit-H3 were detected by immunofluorescence microscope; MPO (red), cit-H3 (green), and DAPI (blue), respectively (magnification, 50×; scale bar, 200μm). Transwell invasion **(F)** and wound healing assays **(G)** were performed to identify the effects of NETs on A549 and SK-MES-1 cells invasion (magnification, 100×) and migration (magnification, 50×). **(H)** Western blot analyzing the expressions levels of EMT markers protein (N-cadherin, E-cadherin, and Vimentin) in A549 and SK-MES-1 cells treated with NETs. (^*^P < 0.05, ^**^P < 0.01).

To investigate whether released NETs are differentially expressed between healthy donors (HD) and NSCLC, we first isolated neutrophils from the blood of HD or NSCLC and observed the cell nuclear morphology by Giemsa staining to estimate neutrophil purity. Under the microscope, we observed the bulk multilobular nucleus of neutrophils (**Figure 1A**). Trypan blue dye exclusion assays revealed that the neutrophil viability was > 97% (**Figure 1B**). We observed that neutrophils isolated from NSCLC patients spontaneously generated NETs, whereas neutrophils from HD produced fewer NETs by immunofluorescence. The capacity of NETs formation from NSCLC patients was higher than HD (**Figure 1C**). We further detected the formation of NETs in NSCLC clinical tissue specimens by immunofluorescence staining. We found that NETs were largely accumulated in the NSCLC tumor tissues, while fewer NETs were formed in normal lung tissues (**Figure 1D**). Activated neutrophils exhibited NETs formation under specific conditions. Freshly isolated neutrophils were evenly seeded in 6-well plates at 1×10^7^/well and stimulated with 100 nM PMA for 4 h. After 4 h of PMA stimulation, light cloud-like substances appeared at the bottom of the 6-well plates. To further confirm whether these structures were NETs, several well-recognized NETs markers, including MPO and cit-H3, were measured by immunofluorescent staining. These markers were more highly expressed after PMA stimulation, and the structures were almost destroyed by treatment with DNase I (**Figure 1E**). In addition, we observed the colocalization of these protein markers with extracellular spider mesh-like DNA around neutrophils.

According to a previous study, the effect of NETs on tumours is not limited to their ability to physically capture cells (37). To explore the potential impact of NETs on the metastasis behaviour of NSCLC, NSCLC cells (A549 and SK-MES-1) were cocultured with PMA-induced NETs, DNase I, or NETs and DNase I together. The invasion assay results showed that NETs significantly promoted the number of invaded NSCLC cells, and these effects were abrogated by DNase I treatment, as the DNA structures of the NETs were digested by DNase I (**Figure 1F**). Moreover, this phenomenon was also observed in wound healing assays; isolated NETs promoted the migration of A549 and SK-MES-1 cells (**Figure 1G**). To investigate the possibility that NETs might induce a switch from epithelial to mesenchymal properties, the expression of EMT marker proteins was evaluated. As shown in **Figure 1H**, NETs promoted the EMT process of NSCLC cells, which was characterized by decreased expression of epithelial markers, such as E-cadherin, and increased expression of mesenchymal markers, such as N-cadherin and vimentin. This effect was suppressed by the digestion of NETs with DNase I (**Figure 1H**). These data suggest that PMA-induced neutrophil NETs formation contributes to the metastatic potential of NSCLC cells.

### LncRNA MIR503HG Expression is Downregulated in NSCLC Cells Stimulated With NETs and is Associated With Poor Survival of NSCLC

**Figure 2:**
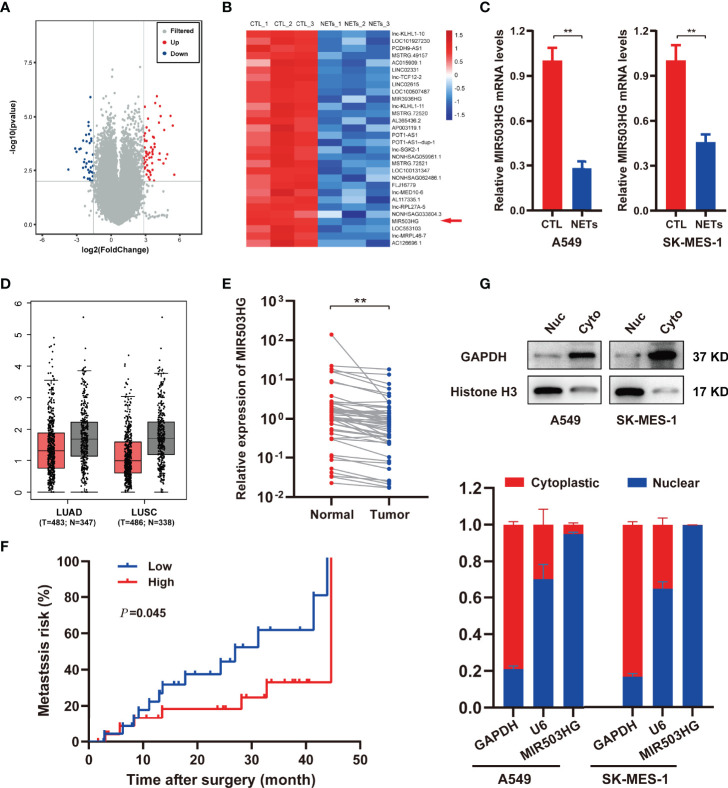
MIR503HG is downregulated in NSCLC cells with NETs stimulation and is associated with poor survival of NSCLC. **(A)** Volcano plot illustrating the differentially expressed lncRNAs in A549 cells treated with or without NETs for 12 h (|log_2_ fold change (FC)| > 2, *P*-value < 0.01). **(B)** Heat map showing the top 30 differentially down-regulated lncRNAs in A549 cells after treatment with NETs for 12 h. Red means up-regulated, blue means down-regulated, separately. **(C)** Relative expression of MIR503HG in A549 and SK-MES-1 cells with or without NETs stimulation. **(D)** MIR503HG is expressed at a lower level in both lung adenocarcinoma (LUAD) and lung squamous cell carcinoma (LUSC) tumor compared to corresponding adjacent normal lung tissues according to the TCGA database. **(E)** The expression of MIR503HG in 50 paired NSCLC tumors and normal tissues were quantified by qRT-PCR. **(F)** Kaplan-Meier analysis of metastasis risk of 50 NSCLC patients divided into two groups based on a middle cutoff of MIR503HG expression. **(G)** MIR503HG is mainly located in the nuclear of NSCLC cells. U6 snRNA (nuclear reserved) and GAPDH mRNAs (exposed to cytoplasm) were used as controls. Data are mean ± SD (n=3). (^**^P < 0.01).

To identify candidate lncRNAs that participate in the pro-metastasis effect of NETs, a transcriptome RNA microarray analysis of A549 cells treated with NETs was performed. We identified 99 differentially expressed lncRNAs (fold change ≥ 4 and *P* < 0.01), of which 59 were upregulated and 40 were downregulated in the NETs-treated group compared to the untreated group (**Figure 2A**). According to bioinformatics analysis, the expression of lncRNA MIR503HG was downregulated in the NETs-stimulated A549 cell group (**Figure 2B**). We investigated the expression of MIR503HG in NETs-stimulated NSCLC cells (A549 and SK-MES-1 cells) by qRT-PCR. Consistently, the expression level of MIR503HG was dramatically lower (*P* < 0.01) in both A549 and SK-MES-1 cells treated with NETs for 12 h than in the control cells (**Figure 2C**), which was consistent with the data generated from our microarray analysis.

To further examine the clinicopathological and prognostic implications of MIR503HG in patients with NSCLC, we next assessed tumour tissue data of NSCLC patients in The Cancer Genome Atlas (TCGA) and demonstrated decreased expression of MIR503HG in both lung adenocarcinoma (LUAD) and lung squamous cell carcinoma (LUSC) tissues compared to the corresponding adjacent normal lung tissues (**Figure 2D**). Then, we analysed the expression of MIR503HG in 50 pairs of NSCLC tissues and corresponding normal tissues from our cohort by qRT-PCR. Our results showed that the expression of MIR503HG was significantly downregulated (*P* < 0.01) in NSCLC tissues compared to the corresponding adjacent normal tissues (**Figure 2E**). The Kaplan–Meier analysis revealed that NSCLC patients with a lower level of MIR503HG had a higher (*P* = 0.045) risk of metastasis (**Figure 2F**). These data indicate that MIR503HG was expressed at low levels in NSCLC tissues or stimulated NETs and might act as a tumour suppressor.

To explore the underlying mechanism by which lncRNA MIR503HG functions in NSCLC, we subsequently explored the subcellular distribution of MIR503HG by subcellular fractionation and qRT-PCR analyses. The results of qRT-PCR, using GAPDH and U6 as controls, showed that MIR503HG was primarily located in the nucleus of A549 and SK-MES-1 cells (**Figure 2G**).

### Overexpression of MIR503HG Substantially Reverses the Metastasis-Promoting Effect of NETs on NSCLC *In Vitro* and *In Vivo*

**Figure 3:**
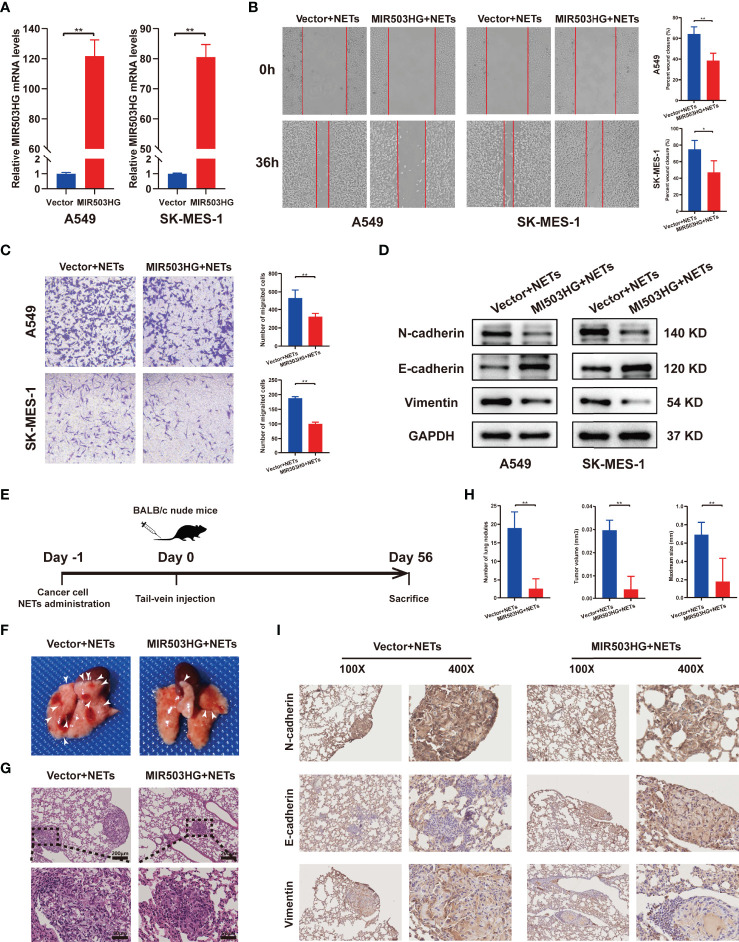
Overexpression of MIR503HG substantially reversed the metastasis-promoting effect of NETs on NSCLC *in vitro* and vivo. **(A)** Expression of MIR503HG was successfully up-regulated in A549 and SK-MES-1 cells. **(B, C)** Wound healing (magnification, 50×) and invasion assays (magnification, 100×) of NSCLC cells that stable transfection of MIR503HG vector versus control vector both treat with NETs 12 h. **(D)** Western blot analysis of the expression of EMT (N-cadherin, E-cadherin, Vimentin) in control and MIR503HG overexpressing A549 and SK-MES-1 cells after NETs treated 12 h. **(E)** Schematic diagram showing the experimental design of the effect of MIR503HG on NETs-induced metastasis. Representative images of the gross lung **(F)** and H&E staining **(G)** of metastatic lung nodules in mice specimens. **(H)** Quantification of the number, volume, and maximum size of metastatic lung nodules. Data are mean ± SD (n=5 nude mice in each group). (magnification, 100×; scale bar, 200 μm, magnification, 200×; scale bar, 50 μm). **(I)** Immunohistochemistry (IHC) detection of N-cadherin, E-cadherin, and Vimentin revealed EMT formation in the NETs-induced lung metastasis model (magnification, 100× and 400×). (^**^P < 0.01).

To fully understand the effect of MIR503HG on NETs metastasis promotion, we further detected the migration, invasion and EMT of NSCLC cells overexpressing MIR503HG. MIR503HG was overexpressed in A549 and SK-MES-1 cells *via* a stable retroviral expression system, and its overexpression was validated by qRT-PCR (**Figure 3A**). We also examined the expression of MIR503HG in normal human bronchial epithelial cell lines (BEAS-2B), NSCLC cells and cells overexpressed. We found that MIR503HG is generally overexpressed in BEAS-2B compared to NSCLC cell lines (**Figure S1A**). Wound healing assays showed that MIR503HG overexpression significantly impaired the NETs-induced migration abilities of NSCLC cells (**Figure 3B**). Similarly, Transwell assays revealed that MIR503HG overexpression inhibited the invasion abilities of cells treated with NETs (**Figure 3C**). Furthermore, the results showed that the expression of the EMT-related epithelial marker E-cadherin was increased but that of the mesenchymal markers N-cadherin and Vimentin was decreased in MIR503HG-overexpressing cells treated with NETs (**Figure 3D**). However, we did not observe significant changes for those markers in siRNA MIR503HG knockdown BEAS-2B cells (**Figures S1B, C**).

To explore the potential inhibitory effect of MIR503HG on NETs-induced NSCLC cell metastasis *in vivo*, MIR503HG-overexpressing NSCLC cells and the corresponding control NSCLC cells were treated with NETs one day before intravenous injection. Then, NETs-treated tumour cells were injected into mice *via* the tail vein, and the mice were sacrificed after 8 weeks (**Figure 3E**). The number of nodules on the surfaces of the lungs was counted (**Figure 3F**), and the presence of metastatic nodules inside the lung and the tumour size were also confirmed by H&E staining (**Figure 3G**). MIR503HG significantly reduced the NETs-induced lung metastasis of cancer cells. We quantified three features of tumours, namely, the number of nodules, tumour volume, and maximum size, to estimate the burden of lung metastasis. The results revealed that all these measures were significantly decreased compared with those in the control mice (**Figure 3H**). Furthermore, the results of IHC staining of tumour tissues suggested that the rate of E-cadherin positivity was increased and N-cadherin and vimentin expression was decreased in the groups with stable MIR503HG expression (**Figure 3I**). All these results suggest that MIR503HG could reverse the facilitation of NSCLC metastasis by NETs both *in vitro* and *in vivo*.

### NETs Promote the Migration and Invasion of NSCLC by Activating the NLRP3 Inflammasome

**Figure 4:**
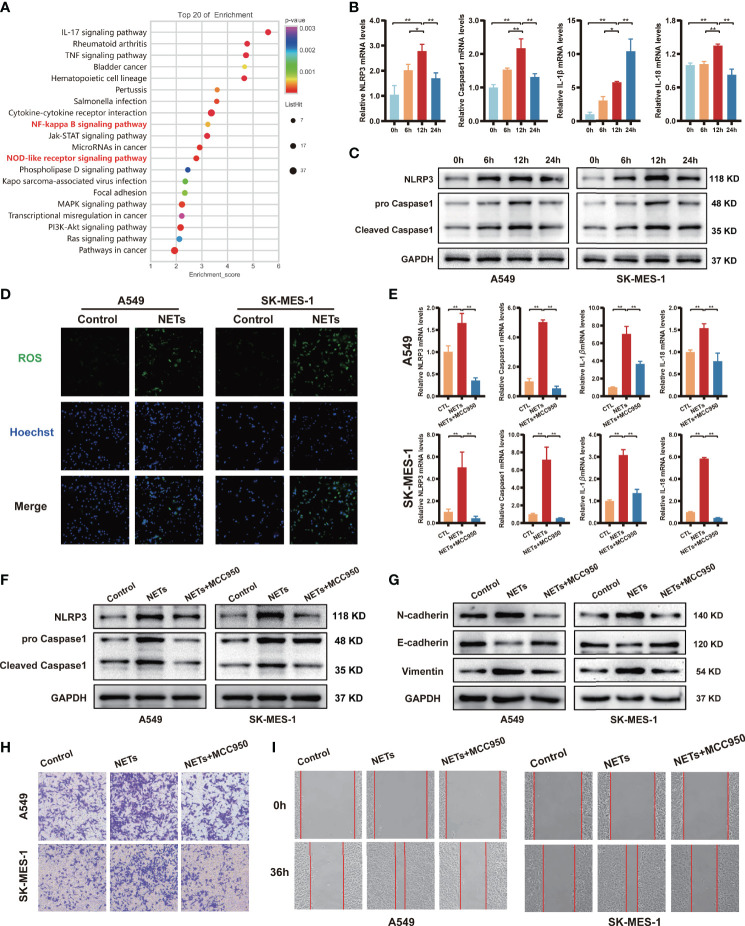
NETs promote migration and invasion of NSCLC by activating the NLRP3 inflammasome. **(A)** Up-regulated NSCLC-related pathways in response to NETs stimulated revealed by KEGG enrichment. **(B)** The mRNA expression of NLRP3, Caspase1, IL-1β and IL-18 in A549 cells was analyzed by qRT-PCR after NETs stimulation at different times. **(C)** Western blotting was used to analyze the expression of NLRP3 and Caspase1 in A549 and SK-MES-1 cells after NETs stimulation for different periods. **(D)** Immunofluorescence was used to observe the expression of ROS in A549 and SK-MES-1 cells treated with NETs for 12 h (magnification, 100×). The expression of NLRP3, Caspase1, IL-1β and IL-18 were detected by qRT-PCR **(E)** and Western blot **(F)** in A549 and SK-MES-1 cells after treating with NETs and NLRP3 inflammasome inhibitor MCC950, respectively. **(G–I)** Estimate the effect of NETs on the level of N-cadherin, E-cadherin, and Vimentin (Western blot) **(G)** in A549 and SK-MES-1 cells and the capacity of invasion (transwell invasion assays; magnification, 100×) **(H)**, migration (wound healing assays; magnification, 50×) **(I)** when inhibiting the expression of the NLRP3 inflammasome by MCC950. (^*^P < 0.05, ^**^P < 0.01).

NETs formation could induce the release of large amounts of cytokines, and inflammasomes are considered key mediators of this process in tumour cells. Nevertheless, inflammasomes have also been shown to suppress the antitumour immune response and play a vital role in tumour cell migration and invasion (38). KEGG pathway analyses of the differentially expressed genes suggested that NETs triggered responses associated with inflammation, including the NOD-like receptor signalling pathway and NF-kappa B signalling pathway (**Figure 4A**). qRT-PCR was performed to validate the RNA microarray data (**Figure 4B**; **Figure S2A**). The expression of a set of NLRP3 inflammasome-associated genes, including NLRP3, Caspase1, IL-1β, and IL-18, was upregulated in a time-dependent manner. NETs stimulation for 12 h induced the most significant increase in the expression levels of NLRP3, Caspase1, and IL-18 compared to the other groups, while IL-1β reached its highest expression levels after NETs stimulation for 24 h (*P* < 0.05). We further validated the protein expression levels of NLRP3 and Caspase1 by Western blotting, and the results were consistent with the qRT-PCR results (**Figure 4C**). Immunofluorescence staining also supported the idea that NETs treatment could increase NLRP3 and Caspase1 expression in NSCLC cells (**Figure S2B**). Compared with the control group, the results of ELISA showed that the secretion level of IL-1β and IL-18 in the NSCLC cells supernatant of the NETs-treated group increased obviously (**Figure S2C**). Then, we used DCFH-DA to examine the effect of NETs on ROS levels in the intracellular. Intracellular ROS levels in both A549 and SK-MES-1 were significantly elevated after 12 h of NETs stimulation (**Figure 4D**). We next examined the role of NLRP3 inflammasomes by treating cells with the NLRP3 receptor-specific small molecule inhibitor MCC950 before NETs treatment. The results showed that MCC950 treatment before NETs treatment significantly decreased the mRNA expression levels of NLRP3 inflammasome-related genes (**Figure 4E**) and decreased the protein expression levels of NLRP3 and Caspase1 (**Figure 4F**). As expected, MCC950 reversed the protein expression levels of EMT-related markers after NETs treatment (**Figure 4G**). In the Transwell and wound healing assays, the number of invasive and migratory NETs-treated NSCLC cells was significantly decreased by treatment with MCC950 compared with the NSCLC cells treated with NETs alone (**Figures 4H, I**). Taken together, these results suggested that NLRP3 inflammasomes contribute to NETs-mediated metastasis promotion in NSCLC.

### NLRP3 Inflammasome Mediates the Effect of MIR503HG on the Inhibition of NETs-Triggered NSCLC Metastasis

**Figure 5:**
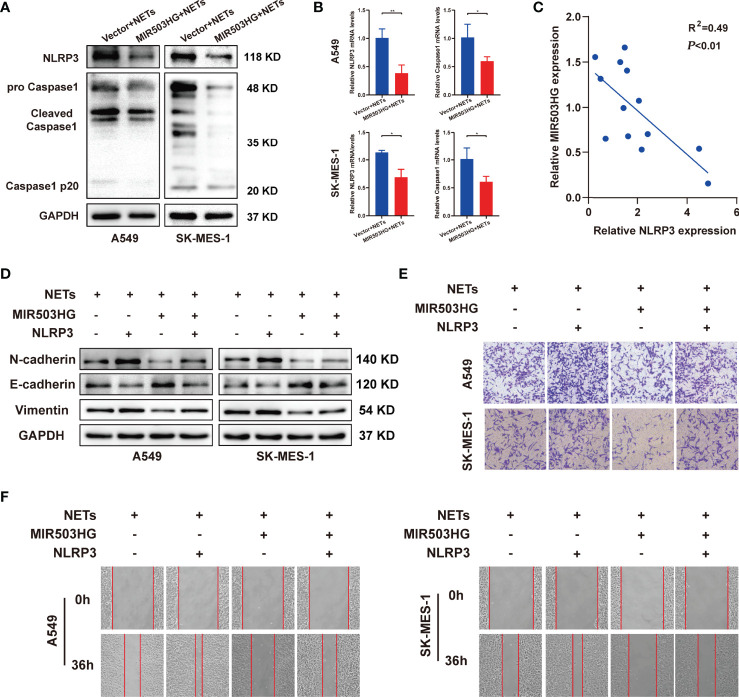
NLRP3 inflammasome mediated the effect of MIR503HG to inhibit NETs-triggered metastasis of NSCLC. **(A, B)** The protein and mRNA expression of NLRP3 and Caspase1 in A549 and SK-MES-1 cells with MIR503HG overexpression were analyzed by Western blot **(A)** and qRT-PCR **(B)** after NETs were stimulated. **(C)** A negative relationship between MIR503HG and NLRP3 in NETs-induced NSCLC cells is presented by correlation analysis. **(D)** Overexpression of NLRP3 attenuated the effect of MIR503HG in inhibiting NETs-triggered EMT in NSCLC cells by Western blot. **(E, F)** Overexpression of NLRP3 effectively reverses the effect of MIR503HG in inhibiting NETs-triggered promotion of NSCLC cells metastasis using transwell assay (magnification, 100×) **(E)** and wound healing assays (magnification, 50×) **(F)**. (^*^P < 0.05, ^**^P < 0.01).

Next, we investigated whether MIR503HG inhibited the NETs-triggered metastasis of NSCLC by affecting the NLRP3 inflammasome. We first examined the protein and mRNA expression levels of NLRP3 inflammasome components in MIR503HG-overexpressing A549 and SK-MES-1 cells treated with NETs. Our Western blotting results revealed that the expression of NLRP3 and Caspase1 was significantly decreased (*P* < 0.05) when MIR503HG was overexpressed in NETs-stimulated NSCLC cells (**Figure 5A**). Examination of NLRP3 and Caspase1 mRNA expression by qRT-PCR also confirmed these results(**Figure 5B**). The mRNA expression of lncRNA MIR503HG was negatively associated with NLRP3 expression in NETs-treated NSCLC cells (**Figure 5C**). A549 and SK-MES-1 cells were transfected with NLRP3-pENTER or empty vector, and the levels of NLRP3 were validated by Western blotting (**Figure S3A**). Then, we transfected NLRP3-pENTER into NSCLC cells stably overexpressing MIR503HG and treated these cells with NETs. NLRP3-pENTER transfection abolished the downregulation of the N-cadherin and vimentin protein levels and the upregulation of the E-cadherin level in the two cell lines overexpressing MIR503HG (**Figure 5D**). Transwell and wound healing assays also indicated that the overexpression of NLRP3 restored the NSCLC cell invasion and migration abilities that had been inhibited by MIR503HG overexpression (**Figures 5E, F**). These results indicated that MIR503HG might indirectly or directly regulate the expression of NLRP3 inflammasome components, and the NLRP3 inflammasome mediated the effect of MIR503HG on the inhibition of NETs-triggered NSCLC metastasis.

### NETs-Induced NLRP3 Inflammasome Activation Promotes NSCLC Progression and is Associated With the Activation of NF-κB

**Figure 6:**
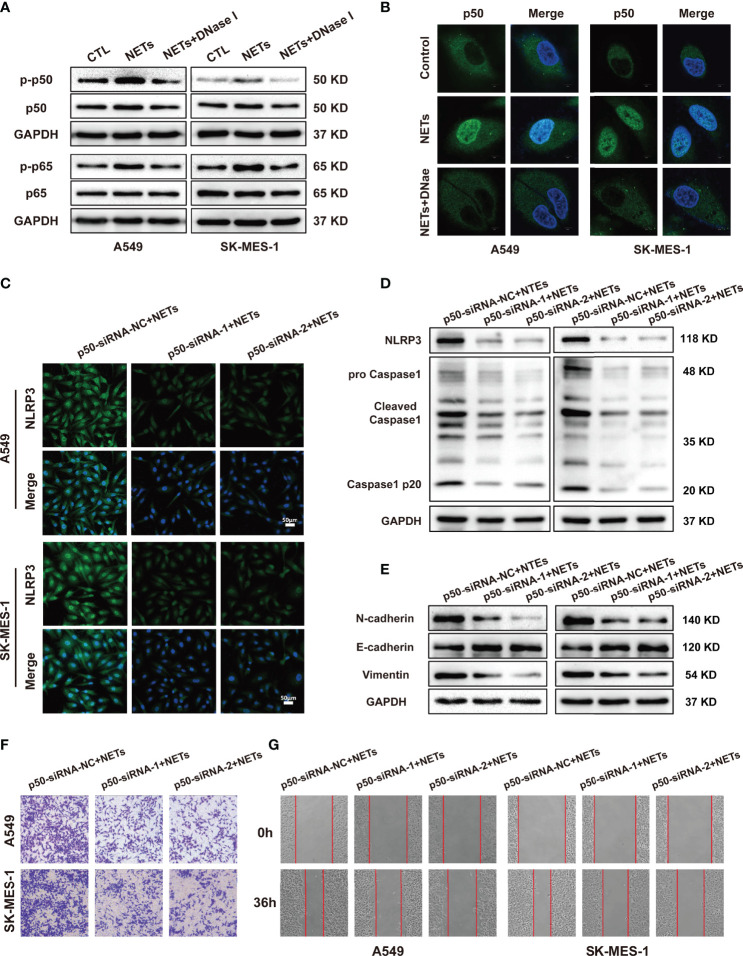
NLRP3 inflammasome induced by NETs promotes NSCLC progression is associated with the activation of NF-κB. **(A)** The protein expression level of p-p50, p50, p-p65, p65 in NSCLC cells treated with NETs was detected by Western blotting. **(B)** Nuclear translocation of NF-κB in A549 and SK-MES-1 cells treated with NETs or combined with DNase I were detected by con-focal microscopy (magnification, 3000×; scale bar, 5μm). Immunofluorescence assays **(C)** and Western blot **(D)** were used to detect the effect of NETs on NLRP3 inflammasome in A549 and SK-MES-1 cells after p50 knockdown (magnification, 200×; scale bar, 50 μm). **(E)** Downregulation of p50 attenuated the effect on promoting EMT of NETs in NSCLC cells by Western blot. **(F, G)** Downregulated p50 reverses NETs-induced promotion of NSCLC cells metastasis using transwell assay (magnification, 100×) **(E)** and wound healing assays (magnification, 50×) **(F)**.

Activation of the NLRP3 inflammasome is associated with the NF-κB signalling pathway. The NF-κB pathway is one of the most important signalling pathways, and this pathway upregulates NLRP3 and pro-IL-1 protein expression (39). In addition, KEGG analysis revealed that the NF-κB signalling pathway ranked in the top 20 pathways in our NETs-stimulated A459 microarray data (**Figure 4A**). To further investigate whether NF-κB was involved in promoting the NETs-induced expression of NLRP3 inflammasome components, we first examined whether the NF-κB signalling pathway was activated in A549 and SK-MES-1 cells after NETs treatment. Our results showed that NETs treatment increased the phosphorylation of NF-κB (p-p50 and p-p65), and this effect was entirely abolished by DNase I treatment (**Figure 6A**). Furthermore, confocal microscopy showed that the translocation of p50 into the nucleus was increased by NETs and inhibited by DNase I (**Figure 6B**). This result indicated that NETs activated the NF-κB signalling pathway in NSCLC cells. To further verify the role of NF-κB in the NETs-induced expression of NLRP3 inflammasome-related proteins, we designed p50-siRNA to knockdown p50 expression in A549 and SK-MES-1 cells and validated the levels of p50 by Western blotting and qRT-PCR (**Figures S3B, C**). P50-siRNA significantly suppressed the expression of NLRP3 and Caspase1 in NETs-stimulated NSCLC cells, as shown by immunofluorescence (**Figure 6C** and **Figure S3D**). Similarly, Western blotting analysis showed that the expression of NLRP3 inflammasome-related proteins in NETs-stimulated NSCLC cells was decreased when the cells were transfected with p50-siRNA compared to the negative control (**Figure 6D**). We further assessed the effect of NETs on the levels of EMT-related proteins and the metastatic ability of NSCLC cells after p50 expression was knocked down. Silencing p50 expression with p50-siRNA reversed the NETs-induced expression of EMT-related proteins (**Figure 6E**). Furthermore, the invasion and wound healing assay results showed that the number of invaded and migrated NSCLC cells after NETs treatment was decreased by the downregulation of p50 expression (**Figures 6F, G**). These results confirmed that NETs triggered the expression of NLRP3 inflammasome components and enhanced the metastatic ability of NSCLC cells *via* activation of the NF-κB signalling pathway.

### MIR503HG Inhibits NETs-Triggered NSCLC Cell Metastasis and NLRP3 Inflammasome Activation in an NF-κB-Dependent Manner

**Figure 7:**
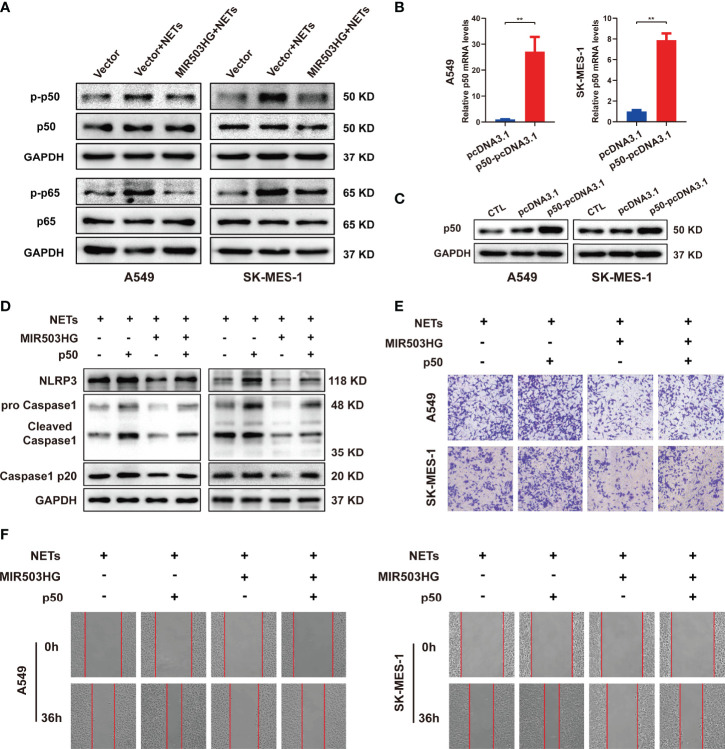
MIR503HG inhibits NETs-triggered NSCLC cells metastasis capacity and NLRP3 inflammasome activation dependently on NF-κB. **(A)** Western blotting was used to detect the changes of p-p50, p50, p-p65 and p65 in MIR503HG overexpression A549 and SK-MES-1 cells after NETs treatment. **(B)** qRT-PCR and **(C)** Western blotting analyses of up-regulating p50 in A549 and SK-MES-1 cells. **(D)** Analysis of the NLRP3 and Caspase1 protein levels in MIR503HG-overexpressed NSCLC cells transfected with p50-pcDNA3.1 and pcDNA3.1 vector by western blot with NETs stimulated. Transwell invasion (magnification, 100×) **(E)** and wound healing assays (magnification, 50×) **(F)** were performed to identify the effects of NETs on MIR503HG-overexpressed NSCLC cells invasion and migration transfected with p50-pcDNA3.1 and pcDNA3.1 vector. (^*^P < 0.05, ^**^P < 0.01).

The above results indicate that downregulation of MIR503HG expression could activate the NF-κB signalling pathway and induce the expression of NLRP3 inflammasome components, which in turn promotes tumour metastasis in NETs-treated NSCLC cells. We further explored the relationship between MIR503HG and the NF-κB/NLRP3 inflammasome *via* regulation of NF-κB expression. We treated MIR503HG-overexpressing A549 and SK-MES-1 cells with NETs and then measured the total and phosphorylated p50 and p65 protein levels by Western blotting. The results indicated that overexpression of MIR503HG decreased the NETs-induced phosphorylation of the NF-κB subunits p50 and p65 in the two NSCLC cell lines (**Figure 7A**). Then, these two NSCLC cell lines were transfected with p50-pcDNA3.1 or empty vector, and the levels of p50 were validated by qRT-PCR and Western blotting (**Figures 7B, C**). We further transfected p50-pcDNA3.1 into NSCLC cells stably overexpressing MIR503HG after NETs treatment. Upregulation of p50 expression abolished the downregulated expression of NLRP3 inflammasome components in NSCLC cells stably overexpressing MIR503HG and treated with NETs (**Figure 7D**). Transwell and wound healing assays also indicated that overexpression of p50 restored the NSCLC cell invasion and migration abilities that had been inhibited by MIR503HG overexpression (**Figures 7E, F**). These data revealed that MIR503HG suppresses the NETs-induced expression of NLRP3 inflammasome components and promotion of metastasis by inhibiting NF-κB activation.

## Discussion

NSCLC metastasis is a very complex multistep process that is closely related to the TME. Increasing evidence suggests that changes in inflammatory cells in the TME play an essential role in the metastasis of tumours (40). Neutrophils are the most abundant inflammatory cells and have been shown to be crucial in tumour progression (6). Tumour-associated neutrophils within the TME are associated with poor prognosis (41). NETs are produced by activated neutrophils and have been confirmed to promote the metastatic dissemination of tumour cells. However, the specific mechanism by which NETs promote NSCLC metastasis remains to be further elucidated. In our present study, we demonstrated the role of neutrophil-secreted NETs in promoting the metastatic capacity of NSCLC by inducing EMT. Mechanistically, NETs induce EMT through the activation of the NF‐κB/NLRP3 inflammasome pathway by downregulating lncRNA MIR503HG expression, ultimately mediating metastasis.

Accumulating evidence has revealed a relationship between NETs and tumour metastasis. Zha et al. suggested that NETs released by tumour-infiltrating neutrophils increase glioma cell proliferation and migration and regulate the microenvironment by modulating HMGB1/RAGE/IL-8 signalling (42). Recent studies have shown that NETs formation after surgical stress promotes liver and pancreatic cancer cell metastasis *via* the production of HMGB1 (14, 43). In our present study, we found that NETs induced by PMA enhanced the migration and invasion of A549 and SK-MES-1 cells, and these effects were abrogated by the NETs inhibitor DNase I. Previous studies have demonstrated that tumour progression and metastasis are associated with the EMT phenotype, which is characterized by the downregulated expression of the key epithelial marker E-cadherin. In contrast, the expression of mesenchymal markers, such as N-cadherin and vimentin, is upregulated (44). Our data showed that E-cadherin expression was downregulated and N-cadherin and vimentin expression was increased by NETs treatment in these two NSCLC cell lines, suggesting that NETs promote NSCLC metastasis by affecting the EMT programme. Our findings are consistent with Zhu et al., who showed that the formation of NETs might be affected by the TME, and NETs-induced EMT is a pivotal event related to dissemination and metastasis in gastric cancer pathogenesis (45). These results indicated the crosstalk between NSCLC metastasis and NETs formation by regulating EMT.

Accumulating evidence has shown that lncRNAs play an essential role in the initiation, progression, and metastasis of various kinds of cancer, including NSCLC (46). At present, no study has reported whether NETs promote tumour progression by affecting the lncRNA transcriptome. Our previous study used microarrays to identify many lncRNAs that are abnormally expressed in human NSCLC cells treated with NETs (28). Here, we revealed that MIR503HG expression is highly deregulated in NSCLC cells stimulated with NETs and that the downregulated expression of MIR503HG facilitated NSCLC cell metastasis *in vitro* and *in vivo*. Initially, MIR503HG was described as a hypoxia-related lncRNA in endothelial cells (47, 48). Several studies have explored the role of MIR503HG in cancer, revealing that MIR503HG inhibits the malignant development of cancer cells by inhibiting proliferation, invasion, and migration (49–51). For the first time, our study revealed that overexpression of MIR503HG substantially reversed the metastasis-promoting effect of NETs on NSCLC, which further suggested that MIR503HG plays a tumour suppressor role in cancers. EMT can be regulated by various factors in different ways, including by TNF-α and TGF-β and through the dysregulation of lncRNA expression (52). Recently, a study reported that MIR503HG inhibits hepatocellular carcinoma cell line metastasis by regulating EMT (51). The present study suggested that MIR503HG reversed the NETs-induced EMT programme, which was consistent with a previous study (53). However, the underlying mechanisms by which NETs promote the EMT programme by affecting MIR503HG expression as well as how NETs enhance the metastatic ability of NSCLC cells remain unclear.

The effects of NETs on tumour metastasis include complex cascades and have been reported to be associated with multiple pathways, including the NF-κB (42, 54), STAT3 (14), MAPK (55), and TLR4/9 pathways (14, 56). NETs, which contain many proinflammatory molecules, have been described as a solid inflammatory stimulus and can stimulate host cells to produce abundant cytokines (57, 58). In light of RNA microarray data, we observed that the expression of a set of inflammatory-associated factors was upregulated in NSCLC cells treated with NETs, which suggested that an aggressive inflammatory response was induced. Through KEGG analysis, the NOD-like receptor and NF-κB signalling pathways were identified as inflammatory-associated events in NETs-triggered metastasis. The NLRP3 inflammasome is a crucial inflammatory factor in the response to pathogens and innate immune stimuli, such as tumorigenesis and development (59). Recent studies have demonstrated that excessive activation of the NLRP3 inflammasome enhances the invasion and metastasis of multiple tumours, including melanoma, hepatocellular carcinoma and pancreatic cancer (59–63). Activation of the NLRP3 inflammasome leads to the release of the proinflammatory cytokines IL-1β and IL-18, which may contribute to cancer development (64). In this study, we reveal a new role for NETs released by neutrophils in the activation and regulation of the inflammasome during the progression of NSCLC. To our knowledge, this is the first time that NETs have been identified as an effective activator of the inflammasome system to promote the malignant development of NSCLC. The current study observed that NLRP3 inflammasomes were activated in NSCLC cells in response to NETs treatment. In contrast, inhibiting the NLRP3 inflammasome led to impaired metastatic cell potential and reversed the EMT programme following increased E-cadherin expression coupled with decreased N-cadherin and vimentin expression. Consistent with our study, Wang et al. also demonstrated that activation of the NLRP3 inflammasome increased the proliferation and migration of NSCLC A549 cells (38). These findings suggest a potential role for NLRP3 activation by NETs in promoting NSCLC. This finding indicates that NLRP3 could be an excellent target for preventing NETs-enhanced metastasis.

Two-signals model of NRLP3 inflammasome activation has been proposed in macrophages (65). The first signal is priming, which is provided by microbial or endogenous molecules that activates NF-κB signalling to promote the expression of NLRP3 and pro-IL-1β, which is a prerequisite for the second signal. The second signal is activation, which is triggered by a variety of stimulation such as ATP, pore-forming toxins and particulate matter that induce events such as K^+^ efflux, generation of ROS and others to initiate NLRP3 inflammasome assembly. Activation of NF-κB was shown to be an intermediate link between NETs and NLRP3 inflammasome activation in NETs-stimulated NSCLC cells. Several reports have revealed that NF-κB mediates the inflammatory response of tumour cells after exposure to NETs, and NETs can activate the NF-κB signalling pathway (42, 54, 66). Growing evidence suggests that NF-κB, a critical transcription factor, is an upstream regulator of NLRP3 and affects the expression of NLRP3 inflammasome components (32, 67–69). Lin et al. revealed that the Platr4 protein interferes with the binding of the NF-κB/Rxrα complex to κB sites, which in turn prevents the transcriptional activation of NLRP3 by NF-κB (69). When NF-κB was blocked, we further found that NETs failed to induce the expression of NLRP3 inflammasome components and enhance the metastatic capacity of NSCLC cells. These findings revealed that the NF-κB/NLRP3 signalling pathway plays a crucial role in NETs-triggered metastasis potential.

Increasing studies have revealed that the particular subcellular localization of lncRNAs usually affects their function; for instance, nuclear lncRNAs can regulate transcription by influencing the activity of transcription factors (70). Yi et al. showed that Gm4419 can directly interact with the p50 subunit of NF-κB and activate the NF-κB pathway (68). Our study revealed that MIR503HG was primarily located in the nucleus. MIR503HG might play a similar role to nuclear lncRNAs in regulating cellular transcription, so we further explored whether the transcription factor NF-κB was regulated by MIR503HG. We found that overexpression of MIR503HG could inhibit NETs-induced NF-κB phosphorylation and NLRP3 inflammasome activation and then suppress the migration and invasion of NSCLC cells, and upregulation of NF-κB expression reversed this effect. MIR503HG might interact with NF-κB to form a nuclear lncRNA-protein complex, which might regulate the downstream NLRP3. These results suggested that MIR503HG inhibited NETs-induced activation of the NF-κB/NLRP3 signalling pathway.

In conclusion, in this study, we demonstrated that NETs promoted NSCLC cell migration and invasion *via* the EMT process. MIR503HG expression was downregulated in NSCLC patient tissues and NETs-treated NSCLC cells. MIR503HG inhibited the NETs-triggered, inflammation-associated metastatic potential of NSCLC cell by inhibiting the activation of the NF-κB/NLRP3 pathway. Our research provides a new mechanism by which NETs function in NSCLC metastasis and identifies novel effective therapeutic targets to treat NSCLC metastasis.

## Data Availability Statement

The original contributions presented in the study are included in the article/**Supplementary Material**. Further inquiries can be directed to the corresponding author.

## Ethics Statement 

The studies involving human participants were reviewed and approved by The Ethics Committee of Medical innovation center of the First Affiliated Hospital of Nanchang University. The patients/participants provided their written informed consent to participate in this study.

## Author Contributions

YW carried out most experiments, analyzed the data and drafted the main manuscript. YW, XYQ, SKY and XWZ participated in the animal experiments. YY conducted the assessment of histopathological changes. SYL and BQF participated in the immunofluorescence and con-focal microscopy experiments. CF and LC drew up part of the manuscript. BY participated in the part of the PCR, WB and ELISA experiments. YL and FL contributed to the study's design, supervision, data analysis and guided writing. All authors read and approved the final version of the manuscript.

## Funding

This study was supported by the grants from National Natural Science Foundation of China (No.81560379, 81460292, 81660315), Surface project of the Natural Science Foundation of Jiangxi Province (No.20181BAB205046, No.20202BAB216031), The Graduate Student Innovation Special Fund Project of Jiangxi Province (No. YC2021-B039).

## Conflict of Interest

The authors declare that the research was conducted in the absence of any commercial or financial relationships that could be construed as a potential conflict of interest.

## Publisher’s Note

All claims expressed in this article are solely those of the authors and do not necessarily represent those of their affiliated organizations, or those of the publisher, the editors and the reviewers. Any product that may be evaluated in this article, or claim that may be made by its manufacturer, is not guaranteed or endorsed by the publisher.

## Supplementary Material

Supplementary Figure 1 **(A)** The expression of MIR503HG mRNA levels in BEAS-2B, up-regulating MIR503HG in A549 and SK-MES-1 cells were analyzed by qRT-PCR. **(B)** qRT-PCR was used to analyze MIR503HG knockdown efficiency in BEAS-2B cells. **(C)** Western blot analyzing the expressions levels of EMT markers protein in BEAS-2B cells after MIR503HG knockdown. (**P* < 0.05, ***P* < 0.01, ns, not significant).

Supplementary Figure 2 **(A)** The mRNA expression of NLRP3, Caspase1, IL-1β and IL-18 in SK-MES-1 cells were analyzed by qRT-PCR after NETs stimulation at different times. **(B)** Immunofluorescence was used to observe the expression of NLRP3 and Caspase1 in A549 and SK-MES-1 cells treated with NETs for 12 h (magnification, 200×; scale bar, 50μm). **(C)** The expression of IL-1β and IL-18 in the supernatant of A549 and SK-MES-1 treated with NETs 12 h was assessed by ELISA. (***P* < 0.01, ns, not significant).

Supplementary Figure 3 **(A)** Western blotting was used to analyze NLRP3 overexpression transfection efficiency in A549 and SK-MES-1 cells. Western blotting **(B)** and qRT-PCR **(C)** were used to analyze p50 knockdown efficiency in A549 and SK-MES-1 cells. **(D)** Immunofluorescence assays were used to detect the effect of NETs on NLRP3 inflammasome in A549 and SK-MES-1 cells after p50 knockdown (magnification, 200×; scale bar, 50 μm). (***P* < 0.01).

Supplementary Table 1 The primers sequences for qRT-PCR analyses.

## References

[1] Siegel RL Miller KD Jemal A. Cancer Statistics, 2020. CA Cancer J Clin (2020) 70(1):7–30. doi: 10.3322/caac.21590 31912902

[2] Chen VW Ruiz BA Hsieh MC Wu XC Ries LA Lewis DR. Analysis of Stage and Clinical/Prognostic Factors for Lung Cancer From SEER Registries: AJCC Staging and Collaborative Stage Data Collection System. Cancer (2014) 120 Suppl 23:3781–92. doi: 10.1002/cncr.29045 PMC4239667 25412390

[3] Ekeke CN Mitchell C Schuchert M Dhupar R Luketich JD Okusanya OT. Early Distant Recurrence in Patients With Resected Stage I Lung Cancer: A Case Series of "Blast Metastasis". Clin Lung Cancer (2021) 22(1):e132–5. doi: 10.1016/j.cllc.2020.09.002 PMC8669737 33144072

[4] Zhuang X Zhang H Hu G. Cancer and Microenvironment Plasticity: Double-Edged Swords in Metastasis. Trends Pharmacol Sci (2019) 40(6):419–29. doi: 10.1016/j.tips.2019.04.005 PMC7518789 31078320

[5] Quail DF Joyce JA. Microenvironmental Regulation of Tumor Progression and Metastasis. Nat Med (2013) 19(11):1423–37. doi: 10.1038/nm.3394 PMC3954707 24202395

[6] Tüting T de Visser KE. CANCER. How Neutrophils Promote Metastasis. Science (2016) 352(6282):145–6. doi: 10.1126/science.aaf7300 27124439

[7] Coffelt SB Kersten K Doornebal CW Weiden J Vrijland K Hau CS. IL-17-Producing γδ T Cells and Neutrophils Conspire to Promote Breast Cancer Metastasis. Nature (2015) 522(7556):345–8. doi: 10.1038/nature14282 PMC4475637 25822788

[8] Coffelt SB Wellenstein MD de Visser KE. Neutrophils in Cancer: Neutral No More. Nat Rev Cancer (2016) 16(7):431–46. doi: 10.1038/nrc.2016.52 27282249

[9] Brinkmann V Reichard U Goosmann C Fauler B Uhlemann Y Weiss DS. Neutrophil Extracellular Traps Kill Bacteria. Science (2004) 303(5663):1532–5. doi: 10.1126/science.1092385 15001782

[10] Fuchs TA Abed U Goosmann C Hurwitz R Schulze I Wahn V. Novel Cell Death Program Leads to Neutrophil Extracellular Traps. J Cell Biol (2007) 176(2):231–41. doi: 10.1083/jcb.200606027 PMC2063942 17210947

[11] Papayannopoulos V. Neutrophil Extracellular Traps in Immunity and Disease. Nat Rev Immunol (2018) 18(2):134–47. doi: 10.1038/nri.2017.105 28990587

[12] Nicolás-Ávila JÁ Adrover JM Hidalgo A. Neutrophils in Homeostasis, Immunity, and Cancer. Immunity (2017) 46(1):15–28. doi: 10.1016/j.immuni.2016.12.012 28099862

[13] Albrengues J Shields MA Ng D Park CG Ambrico A Poindexter ME. Neutrophil Extracellular Traps Produced During Inflammation Awaken Dormant Cancer Cells in Mice. Science (2018) 361(6409):eaao4227. doi: 10.1126/science.aao4227 30262472 PMC6777850

[14] Tohme S Yazdani HO Al-Khafaji AB Chidi AP Loughran P Mowen K. Neutrophil Extracellular Traps Promote the Development and Progression of Liver Metastases After Surgical Stress. Cancer Res (2016) 76(6):1367–80. doi: 10.1158/0008-5472.CAN-15-1591 PMC4794393 26759232

[15] Yang L Liu Q Zhang X Liu X Zhou B Chen J. DNA of Neutrophil Extracellular Traps Promotes Cancer Metastasis *via* CCDC25. Nature (2020) 583(7814):133–8. doi: 10.1038/s41586-020-2394-6 32528174

[16] Teijeira Á Garasa S Gato M Alfaro C Migueliz I Cirella A. CXCR1 and CXCR2 Chemokine Receptor Agonists Produced by Tumors Induce Neutrophil Extracellular Traps That Interfere With Immune Cytotoxicity. Immunity (2020) 52(5):856–871.e8. doi: 10.1016/j.immuni.2020.03.001 32289253

[17] Demers M Wagner DD. Neutrophil Extracellular Traps: A New Link to Cancer-Associated Thrombosis and Potential Implications for Tumor Progression. Oncoimmunology (2013) 2(2):e22946. doi: 10.4161/onci.22946 23526174 PMC3601165

[18] Cools-Lartigue J Spicer J McDonald B Gowing S Chow S Giannias B. Neutrophil Extracellular Traps Sequester Circulating Tumor Cells and Promote Metastasis. J Clin Invest (2013) 123(8):3446-58. doi: 10.1172/JCI67484 PMC3726160 23863628

[19] Aldabbous L Abdul-Salam V McKinnon T Duluc L Pepke-Zaba J Southwood M. Neutrophil Extracellular Traps Promote Angiogenesis: Evidence From Vascular Pathology in Pulmonary Hypertension. Arterioscler Thromb Vasc Biol (2016) 36(10):2078–87. doi: 10.1161/ATVBAHA.116.307634 27470511

[20] Stankovic B Bjørhovde H Skarshaug R Aamodt H Frafjord A Müller E. Immune Cell Composition in Human Non-Small Cell Lung Cancer. Front Immunol (2018) 9:3101. doi: 10.3389/fimmu.2018.03101 30774636 PMC6367276

[21] Oklu R Sheth RA Wong K Jahromi AH Albadawi H. Neutrophil Extracellular Traps are Increased in Cancer Patients But Does Not Associate With Venous Thrombosis. Cardiovasc Diagn Ther (2017) 7(Suppl 3):S140–9. doi: 10.21037/cdt.2017.08.01 PMC5778521 29399517

[22] Lee J Lee D Lawler S Kim Y. Role of Neutrophil Extracellular Traps in Regulation of Lung Cancer Invasion and Metastasis: Structural Insights From a Computational Model. PloS Comput Biol (2021) 17(2):e1008257. doi: 10.1371/journal.pcbi.1008257 33596197 PMC7920364

[23] Rayes RF Mouhanna JG Nicolau I Bourdeau F Giannias B Rousseau S. Primary Tumors Induce Neutrophil Extracellular Traps With Targetable Metastasis Promoting Effects. JCI Insight (2019) 5(16):e128008. doi: 10.1172/jci.insight.128008 PMC6777835 31343990

[24] Kopp F Mendell JT. Functional Classification and Experimental Dissection of Long Noncoding RNAs. Cell (2018) 172(3):393–407. doi: 10.1016/j.cell.2018.01.011 29373828 PMC5978744

[25] Khan S Masood M Gaur H Ahmad S Syed MA. Long non-Coding RNA: An Immune Cells Perspective. Life Sci (2021) 271:119152. doi: 10.1016/j.lfs.2021.119152 33548285

[26] Acha-Sagredo A Uko B Pantazi P Bediaga NG Moschandrea C Rainbow L. Long non-Coding RNA Dysregulation is a Frequent Event in non-Small Cell Lung Carcinoma Pathogenesis. Br J Cancer (2020) 122(7):1050–8. doi: 10.1038/s41416-020-0742-9 PMC7109049 32020063

[27] Schmitt AM Chang HY. Long Noncoding RNAs in Cancer Pathways. Cancer Cell (2016) 29(4):452–63. doi: 10.1016/j.ccell.2016.03.010 PMC4831138 27070700

[28] Fang C Liu F Wang Y Yuan S Chen R Qiu X. A Innovative Prognostic Symbol Based on Neutrophil Extracellular Traps (NETs)-Related lncRNA Signature in non-Small-Cell Lung Cancer. Aging (Albany NY) (2021) 13(13):17864–79. doi: 10.18632/aging.203289 PMC8312458 34257164

[29] Zitvogel L Kepp O Galluzzi L Kroemer G. Inflammasomes in Carcinogenesis and Anticancer Immune Responses. Nat Immunol (2012) 13(4):343–51. doi: 10.1038/ni.2224 22430787

[30] Missiroli S Perrone M Boncompagni C Borghi C Campagnaro A Marchetti F. Targeting the NLRP3 Inflammasome as a New Therapeutic Option for Overcoming Cancer. Cancers (Basel) (2021) 13(10):2297. doi: 10.3390/cancers13102297 34064909 PMC8151587

[31] Wang Z Zhang S Xiao Y Zhang W Wu S Qin T. NLRP3 Inflammasome and Inflammatory Diseases. Oxid Med Cell Longev (2020) 2020:4063562. doi: 10.1155/2020/4063562 32148650 PMC7049400

[32] Ma M Pei Y Wang X Feng J Zhang Y Gao MQ. LncRNA XIST Mediates Bovine Mammary Epithelial Cell Inflammatory Response *via* NF-κb/NLRP3 Inflammasome Pathway. Cell Prolif (2019) 52(1):e12525. doi: 10.1111/cpr.12525 30362186 PMC6430464

[33] Kahlenberg JM Carmona-Rivera C Smith CK Kaplan MJ. Neutrophil Extracellular Trap-Associated Protein Activation of the NLRP3 Inflammasome is Enhanced in Lupus Macrophages. J Immunol (2013) 190(3):1217–26. doi: 10.4049/jimmunol.1202388 PMC3552129 23267025

[34] Hu Q Shi H Zeng T Liu H Su Y Cheng X. Increased Neutrophil Extracellular Traps Activate NLRP3 and Inflammatory Macrophages in Adult-Onset Still's Disease. Arthritis Res Ther (2019) 21(1):9. doi: 10.1186/s13075-018-1800-z 30616678 PMC6323819

[35] Huang W Jiao J Liu J Huang M Hu Y Ran W. MFG-E8 Accelerates Wound Healing in Diabetes by Regulating "NLRP3 Inflammasome-Neutrophil Extracellular Traps" Axis. Cell Death Discovery (2020) 6(1):84. doi: 10.1038/s41420-020-00318-7 32963812 PMC7484765

[36] Najmeh S Cools-Lartigue J Giannias B Spicer J Ferri LE. Simplified Human Neutrophil Extracellular Traps (NETs) Isolation and Handling. J Vis Exp (2015) (98):52687. doi: 10.3791/52687 PMC4541576 25938591

[37] Masucci MT Minopoli M Del Vecchio S Carriero MV. The Emerging Role of Neutrophil Extracellular Traps (NETs) in Tumor Progression and Metastasis. Front Immunol (2020) 11:1749. doi: 10.3389/fimmu.2020.01749 33042107 PMC7524869

[38] Wang Y Kong H Zeng X Liu W Wang Z Yan X. Activation of NLRP3 Inflammasome Enhances the Proliferation and Migration of A549 Lung Cancer Cells. Oncol Rep (2016) 35(4):2053–64. doi: 10.3892/or.2016.4569 26782741

[39] Liu D Yang P Gao M Yu T Shi Y Zhang M. NLRP3 Activation Induced by Neutrophil Extracellular Traps Sustains Inflammatory Response in the Diabetic Wound. Clin Sci (Lond) (2019) 133(4):565–82. doi: 10.1042/CS20180600 30626731

[40] Lambert AW Pattabiraman DR Weinberg RA. Emerging Biological Principles of Metastasis. Cell (2017) 168(4):670–91. doi: 10.1016/j.cell.2016.11.037 PMC5308465 28187288

[41] Gregory AD Houghton AM. Tumor-Associated Neutrophils: New Targets for Cancer Therapy. Cancer Res (2011) 71(7):2411–6. doi: 10.1158/0008-5472.CAN-10-2583 21427354

[42] Zha C Meng X Li L Mi S Qian D Li Z. Neutrophil Extracellular Traps Mediate the Crosstalk Between Glioma Progression and the Tumor Microenvironment *via* the HMGB1/RAGE/IL-8 Axis. Cancer Biol Med (2020) 17(1):154–68. doi: 10.20892/j.issn.2095-3941.2019.0353 PMC7142852 32296583

[43] Kajioka H Kagawa S Ito A Yoshimoto M Sakamoto S Kikuchi S. Targeting Neutrophil Extracellular Traps With Thrombomodulin Prevents Pancreatic Cancer Metastasis. Cancer Lett (2021) 497:1–13. doi: 10.1016/j.canlet.2020.10.015 33065249

[44] Kalluri R Weinberg RA. The Basics of Epithelial-Mesenchymal Transition. J Clin Invest (2009) 119(6):1420–8. doi: 10.1172/JCI39104 PMC2689101 19487818

[45] Zhu T Zou X Yang C Li L Wang B Li R. Neutrophil Extracellular Traps Promote Gastric Cancer Metastasis by Inducing Epithelial-Mesenchymal Transition. Int J Mol Med (2021) 48(1):127. doi: 10.3892/ijmm.2021.4960 34013374 PMC8128417

[46] Luo DB Lv HB Sun XH Wang Y Chu JH Salai AL. LncRNA TRERNA1 Promotes Malignant Progression of NSCLC Through Targeting Foxl1. Eur Rev Med Pharmacol Sci (2020) 24(3):1233–42. doi: 10.26355/eurrev_202002_20176 32096153

[47] Fiedler J Breckwoldt K Remmele CW Hartmann D Dittrich M Pfanne A. Development of Long Noncoding RNA-Based Strategies to Modulate Tissue Vascularization. J Am Coll Cardiol (2015) 66(18):2005–15. doi: 10.1016/j.jacc.2015.07.081 PMC4631810 26516004

[48] Cao X Fan QL. LncRNA MIR503HG Promotes High-Glucose-Induced Proximal Tubular Cell Apoptosis by Targeting miR-503-5p/Bcl-2 Pathway. Diabetes Metab Syndr Obes (2020) 13:4507–17. doi: 10.2147/DMSO.S277869 PMC7691658 33262626

[49] Muys BR Lorenzi JC ZaNETste DL de Barros Lima e Bueno R de Araújo LF Dinarte-Santos AR. Placenta-Enriched LincRNAs MIR503HG and LINC00629 Decrease Migration and Invasion Potential of JEG-3 Cell Line. PloS One (2016) 11(3):e0151560. doi: 10.1371/journal.pone.0151560 27023770 PMC4833476

[50] Wang SM Pang J Zhang KJ Zhou ZY Chen FY. lncRNA MIR503HG Inhibits Cell Proliferation and Promotes Apoptosis in TNBC Cells *via* the miR-224-5p/HOXA9 Axis. Mol Ther Oncolytics (2021) 21:62–73. doi: 10.1016/j.omto.2021.03.009 33869743 PMC8027537

[51] Song S Qiu X. LncRNA Mir503hg Inhibits Epithelial-Mesenchymal Transition and Angiogenesis in Hepatocellular Carcinoma by Enhancing PDCD4 *via* Regulation of miR-15b. Dig Liver Dis (2021) 53(1):107–16. doi: 10.1016/j.dld.2020.09.008 33046427

[52] Lamouille S Xu J Derynck R. Molecular Mechanisms of Epithelial-Mesenchymal Transition. Nat Rev Mol Cell Biol (2014) 15(3):178–96. doi: 10.1038/nrm3758 PMC4240281 24556840

[53] Lin H Wang J Wang T Wu J Wang P Huo X. The LncRNA MIR503HG/miR-224-5p/TUSC3 Signaling Cascade Suppresses Gastric Cancer Development *via* Modulating ATF6 Branch of Unfolded Protein Response. Front Oncol (2021) 11:708501. doi: 10.3389/fonc.2021.708501 34381729 PMC8352579

[54] Yang L Liu L Zhang R Hong J Wang Y Wang J. IL-8 Mediates a Positive Loop Connecting Increased Neutrophil Extracellular Traps (NETs) and Colorectal Cancer Liver Metastasis. J Cancer (2020) 11(15):4384–96. doi: 10.7150/jca.44215 PMC7255375 32489457

[55] Zhou J Yang Y Gan T Li Y Hu F Hao N. Lung Cancer Cells Release High Mobility Group Box 1 and Promote the Formation of Neutrophil Extracellular Traps. Oncol Lett (2019) 18(1):181–8. doi: 10.3892/ol.2019.10290 PMC6540031 31289487

[56] Yang LY Luo Q Lu L Zhu WW Sun HT Wei R. Increased Neutrophil Extracellular Traps Promote Metastasis Potential of Hepatocellular Carcinoma *via* Provoking Tumorous Inflammatory Response. J Hematol Oncol (2020) 13(1):3. doi: 10.1186/s13045-019-0836-0 31907001 PMC6945602

[57] Warnatsch A Ioannou M Wang Q Papayannopoulos V. Inflammation. Neutrophil Extracellular Traps License Macrophages for Cytokine Production in Atherosclerosis. Science (2015) 349(6245):316–20. doi: 10.1126/science.aaa8064 PMC4854322 26185250

[58] Tall AR Westerterp M. Inflammasomes, Neutrophil Extracellular Traps, and Cholesterol. J Lipid Res (2019) 60(4):721–7. doi: 10.1194/jlr.S091280 PMC6446695 30782961

[59] Moossavi M Parsamanesh N Bahrami A Atkin SL Sahebkar A. Role of the NLRP3 Inflammasome in Cancer. Mol Cancer (2018) 17(1):158. doi: 10.1186/s12943-018-0900-3 30447690 PMC6240225

[60] Hu H Wang Y Ding X He Y Lu Z Wu P. Long non-Coding RNA XLOC_000647 Suppresses Progression of Pancreatic Cancer and Decreases Epithelial-Mesenchymal Transition-Induced Cell Invasion by Down-Regulating Nlrp3. Mol Cancer (2018) 17(1):18. doi: 10.1186/s12943-018-0761-9 29386037 PMC5793431

[61] Ahmad I Muneer KM Tamimi IA Chang ME Ata MO Yusuf N. Thymoquinone Suppresses Metastasis of Melanoma Cells by Inhibition of NLRP3 Inflammasome. Toxicol Appl Pharmacol (2013) 270(1):70–6. doi: 10.1016/j.taap.2013.03.027 23583630

[62] Fan SH Wang YY Lu J Zheng YL Wu DM Li MQ. Luteoloside Suppresses Proliferation and Metastasis of Hepatocellular Carcinoma Cells by Inhibition of NLRP3 Inflammasome. PloS One (2014) 9(2):e89961. doi: 10.1371/journal.pone.0089961 24587153 PMC3935965

[63] Karki R Man SM Kanneganti TD. Inflammasomes and Cancer. Cancer Immunol Res (2017) 5(2):94–9. doi: 10.1158/2326-6066.CIR-16-0269 PMC5593081 28093447

[64] Baker KJ Houston A Brint E. IL-1 Family Members in Cancer; Two Sides to Every Story. Front Immunol (2019) 10:1197. doi: 10.3389/fimmu.2019.01197 31231372 PMC6567883

[65] He Y Hara H Núñez G. Mechanism and Regulation of NLRP3 Inflammasome Activation. Trends Biochem Sci (2016) 41:1012–21. doi: 10.1016/j.tibs.2016.09.002 PMC5123939 27669650

[66] Zhu B Zhang X Sun S Fu Y Xie L Ai P. NF-κb and Neutrophil Extracellular Traps Cooperate to Promote Breast Cancer Progression and Metastasis. Exp Cell Res (2021) 405(2):112707. doi: 10.1016/j.yexcr.2021.112707 34153301

[67] Zou J Yang Y Yang Y Liu X. Polydatin Suppresses Proliferation and Metastasis of non-Small Cell Lung Cancer Cells by Inhibiting NLRP3 Inflammasome Activation *via* NF-κb Pathway. BioMed Pharmacother (2018) 108:130–6. doi: 10.1016/j.biopha.2018.09.051 30218857

[68] Yi H Peng R Zhang LY Sun Y Peng HM Liu HD. LincRNA-Gm4419 Knockdown Ameliorates NF-κb/NLRP3 Inflammasome-Mediated Inflammation in Diabetic Nephropathy. Cell Death Dis (2017) 8(2):e2583. doi: 10.1038/cddis.2016.451 28151474 PMC5386454

[69] Lin Y Wang S Gao L Zhou Z Yang Z Lin J. Oscillating lncRNA Platr4 Regulates NLRP3 Inflammasome to Ameliorate Nonalcoholic Steatohepatitis in Mice. Theranostics (2021) 11(1):426–44. doi: 10.7150/thno.50281 PMC7681083 33391484

[70] Chen LL. Linking Long Noncoding RNA Localization and Function. Trends Biochem Sci (2016) 41(9):761–72. doi: 10.1016/j.tibs.2016.07.003 27499234

