# Supplementary figures and images for: Neutrophil Extracellular Traps (NETs) Promote Non-Small Cell Lung Cancer Metastasis by Suppressing lncRNA MIR503HG to Activate the NF-κB/NLRP3 Inflammasome Pathway
Source: Front Immunol. 2022 May 30;13:867516. doi: 10.3389/fimmu.2022.867516 (PMC9190762; doi:10.3389/fimmu.2022.867516)

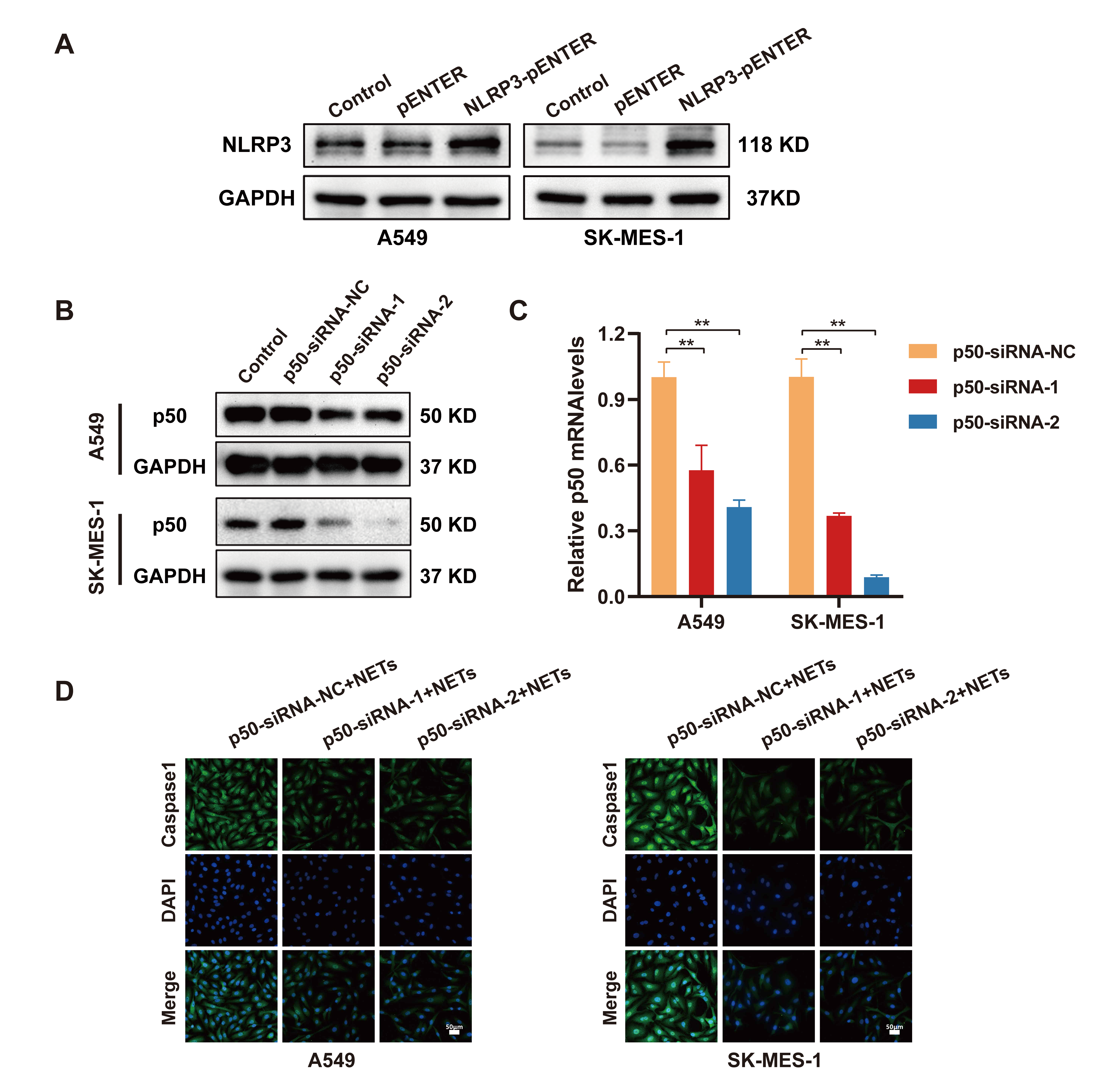

Supplement: Supplementary Figure 3 — (A) Western blotting was used to analyze NLRP3 overexpression transfection efficiency in A549 and SK-MES-1 cells. Western blotting (B) and qRT-PCR (C) were used to analyze p50 knockdown efficiency in A549 and SK-MES-1 cells. (D) Immunofluorescence assays were used to detect the effect of NETs on NLRP3 inflammasome in A549 and SK-MES-1 cells after p50 knockdown (magnification, 200×; scale bar, 50 μm). (**P < 0.01). [file Image_3.tif]
