# Supplementary material for: Neutrophil Extracellular Traps (NETs) Promote Non-Small Cell Lung Cancer Metastasis by Suppressing lncRNA MIR503HG to Activate the NF-κB/NLRP3 Inflammasome Pathway
Source: Front Immunol. 2022 May 30;13:867516. doi: 10.3389/fimmu.2022.867516 (PMC9190762; doi:10.3389/fimmu.2022.867516)
Supplement: Supplementary Table 1 — The primers sequences for qRT-PCR analyses. [file Table_1.docx]

Supplementary Table 1. The primers sequences for qRT-PCR analyses.

|  | **Forward** **primer (5’-3’)** | **Reverse primer (5’-3’)** |
| --- | --- | --- |
| MIR503HG | CCAGCCAGCCTTCCTGAAAG | ACTGGAGATGCTGGATGCCT |
| NLRP3 | GATCTTCGCTGCGATCAACAG | CGTGCATTATCTGAACCCCAC |
| Caspase1 | ATCCGTTCCATGGGTGAAGG | GCCCTTTCGGAATAACGGA |
| IL-1β | CTGAGCTCGCCAGTGAAATG | CATGGCCACAACAACTGACG |
| IL-18 | TGACCAAGGAAATCGGCCTC | ATGGTCCGGGGTGCATTATC |
| p50 | AACAGAGAGGATTTCGTTTCCG | TTTGACCTGAGGGTAAGACTTCT |
| GAPDH | TCTGGAAAGCTGTGGCGTGA | AGCTCTGGGATGACCTTGCC |
| U6 | AGAGAAGATTAGCATGGCCCCTG | GTCGTATCCAGTGCAGGGT |
